# ‘You Understand Me’: Experiences of Peer Mentors Delivering Support for a Mindfulness Intervention to Family Carers of People With Intellectual and Developmental Disabilities

**DOI:** 10.1111/jar.70102

**Published:** 2025-08-24

**Authors:** Alex Gordon‐Brown, Caitlin A. Murray, Nikita K. Hayden, Richard P. Hastings, David Mahon, Samantha Flynn

**Affiliations:** ^1^ Centre for Research in Intellectual and Developmental Disabilities (CIDD), University of Warwick Coventry UK; ^2^ School of Education, University of Sheffield; iHuman, University of Sheffield; Centre for Research in Intellectual and Developmental Disabilities (CIDD), University of Warwick Coventry UK; ^3^ Foundation for People With Learning Disabilities London UK

**Keywords:** family carers, mindfulness, parents, peer support, siblings

## Abstract

**Background:**

Family carers of people with intellectual and developmental disabilities are at increased risk of stress and often face barriers to accessing appropriate supports. Peer support can enhance the effects of well‐being interventions, yet research is limited regarding family carers' experiences within peer support roles.

**Method:**

Semi‐structured interviews were conducted with 10 peer mentors (four adult siblings, six parent carers) paid to support other family carers undertaking an online mindfulness intervention. Interviews were recorded and transcribed. The data were analysed using Framework Analysis.

**Results:**

Peer mentors discussed their motivations, the importance of shared experiences within the mentoring relationships, increased confidence and self‐belief, and learning and growing throughout the mentoring role.

**Conclusion:**

Peer mentors spoke positively, discussing benefits within their personal lives and future employment opportunities. Further research is needed regarding the experiences of mentors who withdrew from the role, as well as fathers, brothers and people from ethnic minority communities.

**Summary**

- Family carers increasingly act as peer mentors delivering support to other family carers, but there is little research evidence about their experiences in such roles.
- Interviews were conducted with adult sibling and parent peer mentors to understand their experiences of supporting other family carers undertaking an online mindfulness programme.
- Mentors enjoyed the role and it developed their confidence, felt meaningful, and maintained their motivation to continue supporting other family carers.
- Future research should focus on understanding the diversity of experiences in these roles, including the perspectives of people from ethnic minority communities, fathers, and brothers.

## 1 Background

Given the additional care roles associated with being a family carer of individuals with intellectual and developmental disabilities, there has been considerable interest in adapting, developing and testing interventions that may help to improve the well‐being of parent carers (Bourke‐Taylor et al. 2021) with an increased interest also in considering the needs of sibling carers (Brennan et al. 2023; Arnold et al. 2012). Self‐guided well‐being interventions may be useful because of ease of access and the opportunity to decide where and when to engage with material (Flynn et al. 2020). In the broader mental health research literature, there is evidence to suggest that adding support from another person to self‐guided mental health and well‐being interventions can enhance effectiveness (Whiteman et al. 2016), including for online self‐guided interventions (Fortuna et al. 2020, 2022).

In the family disability literature, peer support has been identified more generally as beneficial to family carers receiving interventions and supports (Hammarberg et al. 2014; Shilling et al. 2013), playing a valuable role through social identification (Haslam et al. 2016) and the reduction of power dynamics often experienced with professionals (Case 2000). The shared experience peers offer can catalyse the ability to speak openly due to the perceived safety and non‐judgemental environments as well as encourage recipients of support to learn from their peers (Shilling et al. 2013). In addition, there is some evidence to suggest that peer support added to an evidence‐based intervention for family carers can increase effectiveness (Bjornstad et al. 2021; Flynn et al. 2020). However, there is a paucity of research on family carer peer mentors' experiences within these support roles, including the experiences of adult siblings.

A small amount of existing research has indicated that family carer peer mentors may themselves benefit from taking on a role in the context of interventions to support others. In their qualitative exploration of peer experiences providing a 1–1 befriending service for families of children with disabilities, Shilling et al. (2015a) and Shilling et al. (2015b) found that befrienders benefited from training, mutual support, and perceptions of helping others as well as expanding their social network. However, negative consequences of additional emotional burden for the befrienders as well as concerns around job performance and the time commitment required were also expressed. Family carer facilitators interviewed about their role co‐delivering Early Positive Approaches to Support (E‐PAtS) recognised a reduction in isolation through providing peer‐based support and highlighted how valuable shared experience was to providing support for others, enabling increased empathy and greater flexibility within sessions to discuss issues raised by family carers whilst also maintaining fidelity to the sessions (Coulman et al. 2022; Coulman et al. 2021). The E‐PAtS intervention was delivered in groups, and so the mix of individuals meant that the level of this shared experience varied and could impact the perceived level of group cohesion. Peer facilitators also acknowledged how comprehensive training and sufficient preparation time played an important role in the development of the necessary confidence and skills prior to undertaking their peer facilitation role (Coulman et al. 2022; Coulman et al. 2021).

The experiences of peer supporters have been explored more widely outside of the field of intellectual and developmental disabilities. For example, Theurer et al. (2022) found in their interviews with 48 peers supporting elderly residents in various settings that peers felt they developed confidence, expanded their social network, and helped themselves by helping others. Additionally, Ahmed et al. (2015) found that peer supporters within the mental health field felt their employment facilitated clinical and psychosocial benefits regarding their own well‐being and recovery, alongside the contribution their role made to their own feelings of hope, empowerment, competence and social engagement within themselves. Ahmed et al. also found supporters felt poorly compensated and expressed frustrations around limited employment opportunities, difficulties maintaining personal well‐being, and the emotional stress of their role. Further research is required to investigate whether these finding are relevant for peer supporters within the field of intellectual and developmental disabilities.

The purpose of the present study was to understand the experiences of the family carers (both parents and adult siblings) who were trained to deliver individual peer mentoring support to other family carers who were taking part in an online mindfulness intervention.

## 2 Methods

### 2.1 Peer Mentor Role

#### 2.1.1 Peer Mentor Recruitment

Peer mentor recruitment was undertaken via project charity partners (i.e., the Foundation for People with Learning Disabilities, Contact, Sibs, and Family Fund) through a combination of advertisements and existing contacts. Prospective peer mentors were asked to complete a short expression of interest form to support shortlisting for interviews. Fifteen family carers (*n* = 10 parents, *n* = 5 adult siblings) were recruited following interviews, trained, and employed as paid peer mentors. Peer mentors were employed and paid by the Foundation for People with Learning Disabilities. Five mentors (*n* = 4 parents, *n* = 1 adult sibling) withdrew at different points early in the process due to familial circumstances, with only one of these five peer mentors mentoring any family carers before withdrawing. Based on previous research (e.g., Flynn et al. 2020), peer mentor withdrawals were anticipated, so we purposefully over‐recruited peer mentors for this project.

#### 2.1.2 Peer Mentor Training and Supervision

A one‐and‐a‐half‐day online training course was hosted on Microsoft Teams by the Foundation for People with Learning Disabilities. Eleven of the recruited peer mentors attended both training days, and the other peer mentors who were unavailable attended catch‐up training sessions with the trainers instead. The online training covered information about the research project, Be Mindful Online (the online mindfulness programme that peer mentors would be mentoring family carers to engage with), safeguarding, and the mentoring role, including understanding and maintaining boundaries, introducing the mentoring manual, the GROW [Goals, Reality, Options, Way Forward] model (Whitmore 1996), and being given ample opportunities to practise peer mentor telephone calls. Peer mentors also had the opportunity to ask questions about the remit of their role. They were all sent the Peer Mentor Manual to support their familiarity and practice opportunities before they began mentoring family carers.

Peer mentors also completed Be Mindful Online before mentoring family carers, so that they were familiar with the intervention that they were mentoring family carers to engage with. Be Mindful Online is a publicly available ten‐session programme, based on mindfulness‐based cognitive therapy. The ten online sessions can be completed in as few as four weeks, with video and audio instructions and exercises, 12 assignments to practise, and six downloadable course handouts. A more detailed overview of Be Mindful Online can be found in Flynn et al. (2020).

Peer mentors were supervised by the Foundation for People with Learning Disabilities throughout the project. Peer supervision was provided by a lead parent mentor who had previously worked with the Foundation for People with Learning Disabilities in a similar capacity, and a lead sibling mentor who had relevant experience that became apparent during the interviews. Formal peer supervision occurred on an ad hoc basis when mentors needed advice or support, and informal peer support was facilitated through WhatsApp group chats.

#### 2.1.3 Peer Mentor Activities

To fulfil their mentoring role, all peer mentors were provided with a mobile telephone. They offered three 30‐minute telephone support calls to family carers who were accessing Be Mindful Online. The peer mentoring was delivered according to a previously co‐produced manual (see Flynn et al. 2020), which was updated to reflect the inclusion of adult sibling carers in addition to parents.

The focus of the support calls was to encourage family carers to start and continue with Be Mindful Online; for example, by exploring ways to protect the time needed to complete it, and to problem‐solve barriers they may be experiencing. Three peer mentors withdrew before being allocated any family carers. Twelve peer mentors were allocated between 3 and 13 family carers during their employment (an average of 8). Eleven peer mentors engaged in family carer mentoring and mentored between 2 and 11 (an average of 6), with those who mentored fewer family carers tending to be those who either ended their employment on the project early or began later.

### 2.2 Participants

Of the ten family carers invited to interview (*n* = 6 parents, *n* = 4 siblings; *n* = 1 male, *n =* 9 female) all agreed to take part. The five peer mentors who withdrew from the project had not provided their consent to be contacted further about an interview and were therefore not invited. This is considered to be an appropriate sample size to gather sufficient data in a qualitative interview study (e.g., Hennink and Kaiser 2021; Braun and Clarke 2013), particularly considering the homogeneity of the sample and the narrow focus of the study (Hennink and Kaiser 2021). Further, it is important to note that no new concepts were being raised by interviewed peer mentors during the final interviews.

Demographic data were gathered for peer mentors about their relationship to the person with intellectual or developmental disability for whom they cared, and their gender. Other demographic data were not required for the purposes of the research project, so were not collected. These data are summarised in Table 1 along with data about their mentoring activities, and the pseudonyms used for peer mentors within this paper.

**TABLE 1:** Peer Mentors demographics and mentoring activities.

Pseudonym	Role	Gender	Number of family carers (allocated) and mentored	Mean number of peer mentoring sessions completed (max. 3)	Length of employment (months)
Grace	Parent Mentor	Female	(8) 5	1.63	7
Beth	Parent Mentor	Female	(11) 5	1.09	13
Paula	Sibling Mentor	Female	(13) 11	2.31	13
Nora	Parent Mentor	Female	(10) 9	2.2	13
Lucy	Sibling Mentor	Female	(9) 8	2.44	13
Eva	Parent Mentor	Female	(3) 3	2.33	9
Jennifer	Parent Mentor	Female	(9) 5	1.11	13
Katie	Sibling Mentor	Female	(11) 9	2.09	13
Sara	Parent Mentor	Female	(11) 6	1.09	13
Angus	Sibling Mentor	Male	(7) 7	2.86	13

### 2.3 Procedure

Peer mentors who had not withdrawn were initially approached by the Foundation for People with Learning Disabilities to ask them if they were happy for their name and contact details to be shared with the interviewer. The interviewer had not previously met or worked with any of the peer mentors and did not have an existing relationship with the Foundation for People with Learning Disabilities to ensure that the interviewer maintained independence. Upon making contact with the peer mentors, the interviewer sent them an information sheet and consent form by email.

Following fully informed consent being obtained, semi‐structured interviews were conducted by AGB via Microsoft Teams between July and September 2023. The interview topic guide contained questions about: mentors' experiences through their recruitment and training, undertaking Be Mindful Online themselves, delivering the peer support, and any wider impact this may have had. The topic guide is included as Supporting Information A. Interviews had a mean length of 49.5 min (range 23–71 min). Interviews were recorded and transcribed verbatim by AGB using the Microsoft Teams transcript function and reconciling this line by line referring to the video/audio recording.

Ethical approval was granted by the University of Warwick's Humanities and Social Sciences Research Ethics Committee (reference 101/21–22).

### 2.4 Data Analysis

Data were analysed using Framework Analysis (Ritchie and Spencer 1994). The first stage involved transcribing and then reading the transcripts for data familiarisation. The initial thematic framework was based on an existing framework from a similar study (Flynn et al. 2020) and was amended to ensure that it was guided by the interview topic guide and study research aims. Indexing was then completed, whereby AGB used NVivo software (version 1.7.1) to apply the framework to the transcripts. Secondary coding was undertaken by SF, and discrepancies in the use of the framework were identified by AGB using the coding stripes function in NVivo, allowing for a line‐by‐line comparison. All differences were discussed to establish where any changes to the framework and/or its use were needed ahead of its continued use by AGB.

A data matrix (or chart) was extrapolated into Microsoft Excel to enable AGB to complete the mapping and interpretation of data. Our approach to interpreting these data took a largely constructionist approach, in that interpretations were grounded in the data and meaning was made from them, whilst acknowledging that our conceptualisation of the data would impact the way in which the interpretation occurred. To check interpretations of the data, SF was involved in the conceptualisation stage, providing challenge and prompting discussion about early interpretations of the data until a final model was developed and agreed by the wider research team. This process was intended to add reliability to the interpretation of the data.

## 3 Results

**FIGURE 1:**
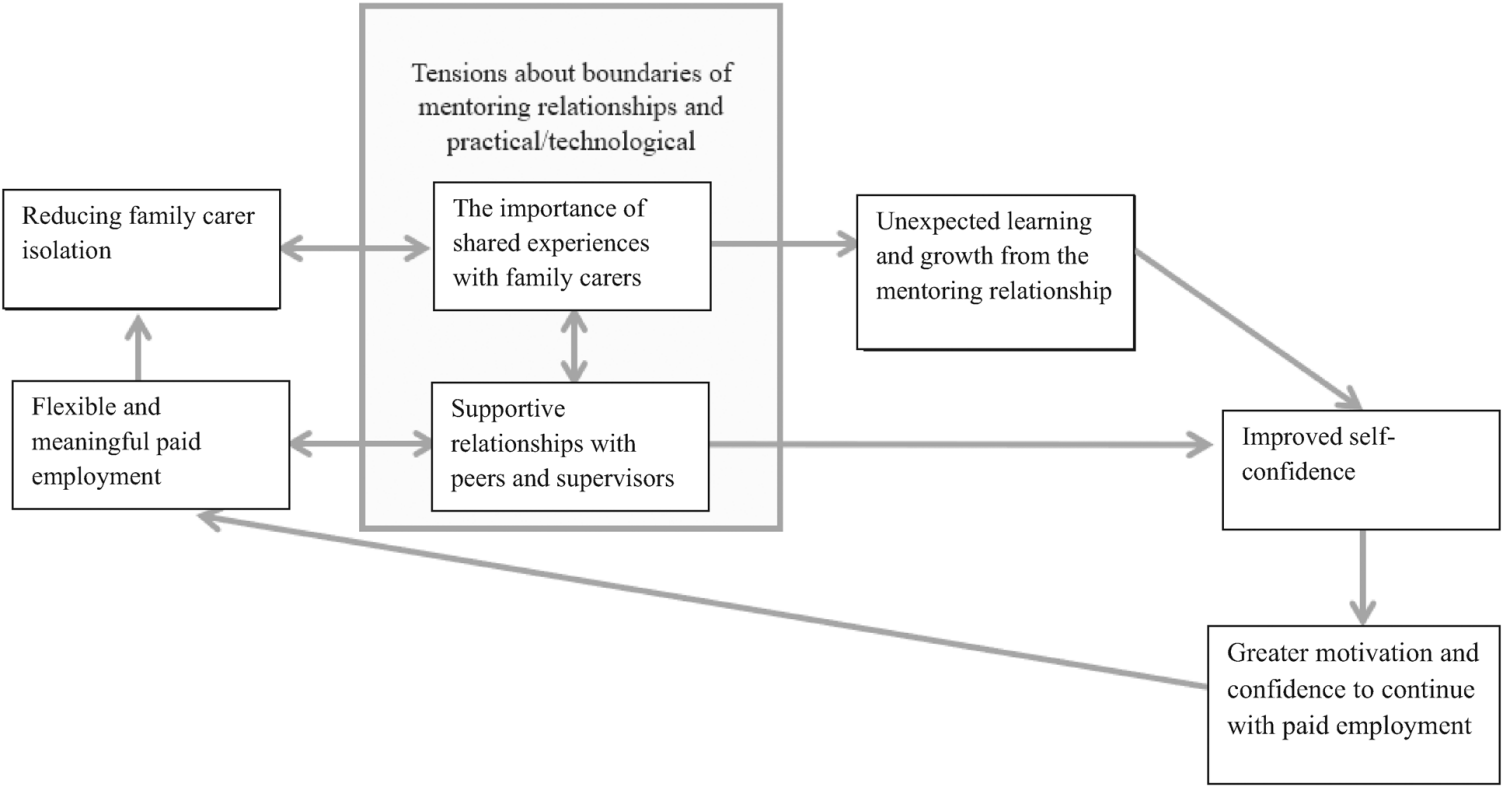
A Visual Summary of Key Themes of the Experiences of Peer Mentors.

Peer mentors discussed how family carers can enter ‘survival mode’, where they can become unable to pause to acknowledge and address the personal impact of their caring role. This feeling, related to reducing family carer isolation, and being employed in a flexible and meaningful paid role were key motivators for peer mentors applying for the role. Their expertise as family carers was a key feature of their role as peer mentors as their shared experience was integral to developing mentoring relationships, and in developing supportive relationships with their fellow peer mentors. As peer mentors progressed through their roles, their self‐confidence organically developed as their experience grew and as they experienced unexpected learning and growth within their role. This led to an increased motivation and confidence to continue with paid employment. Based on the analysis, we developed a visual summary of the overall experiences of peer mentors (Figure 1). This visual summary also includes tensions about the boundaries of the peer mentoring relationship and practical difficulties that may interfere with peer mentors' positive experiences, as raised in shared experiences with family carers.

### 3.1 Peer Mentor Motivation

Peer mentors expressed that, having themselves experienced isolation as a family carer, they wanted to ***reduce isolation for other family carers*** through connecting with others in a similar position to them, and this was an important motivator for applying to the role. Some peer mentors described a desire to give back to others some of the support that they themselves had received previously. This desire to give back suggests that peer mentors motivations for taking on the peer mentoring role were, in part, altruistic.

It's just important to me to support people and help them feel that they're not alone and that there are others that are going through this (Beth, Parent Peer Mentor)

Parent mentors tended to have had more experiences than sibling carers of accessing support from services or other parents, but they described these supports as often fraught with difficulties. Sibling peer mentors noted the scarcity of opportunities to connect with other sibling carers, thus connection with other siblings was critical as a motivator.

When you meet another sibling …you're like ‘oh wait, you understand me’, when we have shared things and it got all these things I've never talked to anybody about (Paula, Sibling Peer Mentor)

Peer mentors appreciated that the role remote with flexible hours, and that it placed value on their experience as a family carer. These factors were motivational to undertaking the peer mentorship role as it allowed them to fit this ***flexible and meaningful paid employment*** around their existing commitments.

I haven't worked for a long time, well I have worked but not getting paid to do anything. And I just thought it would just be really lovely, and helping people too because I'm kind of doing that anyway in a different way (Jennifer, Parent Peer Mentor)

### 3.2 Shared Experiences With Family Carers

Peer mentors felt that the shared understanding and experiences that arose from successful matching with family carers meant that inter‐personal connections were more easily established during the telephone calls, allowing family carers to express themselves. These shared experiences further reinforced the need for meaningful peer connection between peer mentors and family carers.

As soon as my mentees [family carers] knew that I was also a sibling carer… they opened up to me more. And I think they just felt there was that real connection you know and there was definitely a connection … and I think that's a really important aspect of the whole programme (Lucy, Sibling Peer Mentor)

Shared experience of completing Be Mindful Online was considered relationally important by mentors, enabling recognition of potential barriers to engagement for family carers and increasing their ability to provide bespoke problem solving during mentoring calls based on their own experiences of engaging with the intervention. Peer mentors also found that their role provided a sense of accountability, but also offered permission and encouragement for family carers to prioritise themselves. Given that the Be Mindful Online intervention is not family carer specific, peer mentors found that their role in supporting other family carers often gave a different lens for the intervention to be viewed through by mentors and family carers.

I found that it made me focus on the stress of being a sibling. Whereas I've just done mindfulness things and been like ‘oh, you know, need to reduce your stress’. This [peer element of the intervention] mainly focusses on the stress of being a sibling and then talking to other siblings […] It's almost like it's addressing that bit of stress and helping with that (Paula, Sibling Peer Mentor)

Peer mentors had a good understanding of the competing pressures faced by family carers, so could provide non‐judgmental problem‐solving opportunities for family carers when they were expressing negative feelings about not progressing with the intervention.

I think quite a big part of my sort of conversations with people was around the fact that actually, no, you don't have to feel guilty… you know, kind of trying to help people manage their feelings about not being as far ahead as they thought they ought to be. That was quite a big part of it for me, really (Beth, Parent Peer Mentor)

Whilst generally positive, the shared experiences between mentors and family carers sometimes led to tensions arising for mentors about the value and boundaries within the mentoring relationship. Mentors were occasionally unsure about how much of their shared experiences should be raised within the mentoring calls. Mentors also sometimes expressed frustrations about the limitations of the role, as they wanted to offer a deeper relationship than the prescribed mentoring relationship allowed for, to further reduce the isolation felt by family carers by sharing more with their family carer mentees. This occasionally led to questions about the value of having shared experiences with family carers.

But occasionally I was like [we] haven't really talked about our sibling stuff, at all, it was just [we] talked about the mindfulness course when you're like, when is that benefit of me being a sibling, you know. And yeah but then there's other calls …[it was] really useful being a sibling, so that that sibling bit is sometimes a bit like, OK, where this is fitting (Paula, Sibling Peer Mentor)

Despite the understanding that a structured approach was needed to maintain intervention fidelity, boundaries, and family carer expectations of the peer mentor role, some mentors found that strictly following the mentoring manual could remove some of the authenticity of the conversations and undermine the human connection that they felt was needed by family carers.

They needed this connection, you know they needed, they're tired, they're exhausted, they have lots of things around and they needed someone to listen… Sometimes I had, like, more than 30 minutes… These calls that were more interesting and joyful for me than the formal one that we stick to the script and get the target and reminders (Sara, Parent Peer Mentor)

### 3.3 Supportive Relationships With Peers and Supervisors

Peer mentors valued the opportunity for supervision with the lead sibling mentor or parent mentor.

I mean, [lead parent mentor/supervisor] lovely, she's just really, really supportive and obviously she's a parent carer herself, so she gets it, she knows, you know, sort of where the parent carers are coming from. (Beth, Parent Peer Mentor)

Peer mentors also reported valuing their relationships with their fellow peer mentors. The peer mentor WhatsApp groups provided a space to talk about their families, share difficulties within their mentoring roles, and provided an opportunity for peer mentors to express themselves amongst supportive peers. However, some peer mentors who had missed the initial group training, and were instead trained in an individual session, felt as if they were on the periphery of the group and less able to participate in the mentor WhatsApp groups, creating a perception of isolation even amongst peers.

I felt that they already knew each other pretty well. They always responded nicely, but I never felt fully part of the community (Eva, Parent Peer Mentor)

Peer mentors appreciated the practical support from the wider project team, but when there were practical issues that could not be solved readily by the project team (e.g., with uncertainty about contract lengths, difficulties being set up on payroll, unfamiliar technology) mentors could feel frustrated. The supervisory relationship with the project team was fundamental to supporting the development of mentors' self‐belief, particularly those who initially felt more uncertain, which seemed to further motivate mentors and validate their abilities.

So to be told that I was still able to kind of put myself across in a way that was useful and strong, that was really good for me (Beth, Parent Peer Mentor)

### 3.4 Unexpected Learning and Growth

Peer mentors recognised that they were able to bring their experiences and learning to the peer role but also that they learned and grew as a result of the exposure to Be Mindful Online and the peer mentoring role. Mentors described valuing the skills learned from Be Mindful Online in acknowledging the impact that caring has had on their lives and that this acknowledgement better enabled them to support family carers in their mentoring telephone calls. Mentors discussed experiencing a shift in mindset throughout the process, and this was attributed in part to mentors being able to process and reinforce their own learning during mentoring telephone calls.

After having spoken to all these mentees [family carers] as well, going through and all the things that they said it just, yeah, it just almost changed my mindset doing it…but I didn't have someone to talk to while I was doing it and I think it's almost like it's useful talking to someone about it as you go through because it helps you kind of process it (Paula, Sibling Peer Mentor)

Mentors also described experiencing ‘bidirectional’ learning through their calls in a way that was unintentional and unexpected. Problem solving collaboratively allowed mentors to draw on strategies suggested and implemented by family carers in their own lives as well:

She taught me something very important. Like her life is hectic, she's working around the clock. She and her partner managing this whole crazy situation…So it's really nice because she find[s] her way how to do that, how to prioritise herself and do something for herself (Sara, Parent Peer Mentor)

Through consistently encouraging family carers to prioritise themselves and their self‐care, and to maintain boundaries to enable this, mentors found that they were frequently reflecting on this message, and it appeared to help them integrate these strategies into their own lives.

### 3.5 Improved Self‐Confidence

Peer mentors described how their self‐confidence increased throughout the experience, and they outlined that this belief in themselves coincided with the recognition that their skills and experience as a family carer were valuable.

It really boosted me, me in a person because I needed that confidence back in myself, cause I was really depressed and, and, you know, felt that I was the only one (Nora, Parent Peer Mentor)

The flexible nature of the post also allowed mentors who had been out of paid employment for an extended period while focusing on their caring role to take a graded approach to returning to paid employment, allowing them to develop confidence progressively. That the mentoring role was paid was an important factor in peer mentors building self‐confidence, as it gave some external validation that they and their expertise were valued.

It was really helpful because it's like boosted my self‐confidence that I can do it although, although I was at home like taking care of my family for 12 years now, I haven't been in work field for, for ages, that's for me for ages, but I can do it like. Yeah, it, it, it's really helped me gain my confidence again yeah (Sara, Parent Peer Mentor)

The mentoring manual, although previously described as being potentially limiting when developing relationships with family carers, provided a supportive scaffold for mentors. They were able to use this ‘scaffold’ to initially reduce their anxiety and give them opportunities to improve their confidence in their role while it was still relatively unfamiliar. As mentors gained more experience, they organically developed confidence and skills in balancing the relationship with the family carer and the boundaries of the role, becoming less reliant on the mentoring manual as they progressed through their mentoring calls.

So as you go on further on I said you know you build your confidence, you probably can word things slightly differently erm you know you know what works for yourself but having that there just to know that…I'm doing it right (Lucy, Sibling Peer Mentor)

Confidence in the benefit of the role itself also seemed to develop, with mentors expressing that they felt valuable for helping family carers to complete Be Mindful Online. This value reinforced the sense from peer mentors that they were engaged in meaningful employment.

I think that's what I want to hear from, you know that I have made a difference and all of them have said that they feel listened to, feel that someone understands we've got similarities. I think for me, as I mentor, I feel I've done my job…I feel I really feel happy that I was part of it (Nora, Parent Peer Mentor)

Overall mentors reported having overwhelmingly positive experiences of peer mentoring and were motivated to continue the role should the opportunity be available. Mentors experienced ***greater motivation and confidence to continue with paid employment*** following their peer mentoring role. Mentors learned about further employment opportunities through the project team, or informally through the mentor WhatsApp groups. Several mentors reported being able to secure future employment indirectly through their involvement in this project.

…it's brought up a lot of other opportunities that I didn't necessarily think that it would (Beth, Parent Peer Mentor)

## 4 Discussion

Peer mentors described their experiences of mentoring as being overwhelmingly positive, enjoyable and meaningful, and they perceived that they had made a difference to the family carers they supported. A key aspect of the mentoring relationship was the shared understanding and experiences of the mentors and family carers, which facilitated the relationships but could also give rise to difficulties in recognising and maintaining boundaries with family carers. Mentors valued paid work in a meaningful role, regardless of their previous employment history, and this, alongside developing new skills and personal growth, was one of the key mechanisms to increasing their confidence. These findings regarding confidence development are concordant with Theurer et al. (2022) who also found that a structured approach to mentoring and supporting isolated peers is a means of helping mentors feel confident.

Findings around the importance of shared experiences and the importance of ongoing support and supervision for mentors are consistent with findings from Shilling et al. (2015a) and with systematic review findings from Shilling et al. (2013) that mentors can experience positive outcomes when delivering 1–1 support through training, mutual support, and the feeling that they were helping others. The findings from this study underpin both the crucial role that peers can play in supporting family carers and also the added value that family carers can experience by getting involved in formal peer support roles.

Peer mentors expressed their perceptions that experiential overlaps enabled family carers to discuss their struggles and gain empathic acceptance; emphasis on the importance of experiential similarity and its perceived importance to creating empathy and understanding for both parties has also been stressed in other peer support research (Suitor et al. 1995; Veith et al. 2006). The current study extends these findings, postulating benefits for the mentors themselves through these mechanisms. An unexpected benefit for mentors was ‘bidirectional’ learning throughout the support calls. Mentors were able to acquire new strategies for use within their caring roles and reinforce their mindfulness skills as they mentored family carers. These new strategies allowed peer mentors to recontextualise their caring roles, their mindfulness skills, and express gratitude towards the family carers who inadvertently provided reciprocal peer support to the peer mentors.

Some processes were highlighted that mentors felt were limiting the value they could provide within their role. Peer mentors' expressions of frustration at the lack of depth and the structured nature of their peer mentor roles suggest that this may undermine interpersonal relationships due to the emphasis on goal‐directed mentoring tasks rather than on the peer element. These findings around the balancing of peer identity and structured approach are in line with issues discussed within the mental health field regarding the maintenance of the peer component when undertaking peer supported interventions, as formalisation of the role through an institution or perceptible professionalism could undermine and reduce the significance of shared experience (Adams 2020; Simpson et al. 2018). Future research about peer mentoring and peer support for family carers of people with intellectual and developmental disabilities could explore further this perceived tension between maintaining boundaries in a structured mentoring approach and sharing experiences with family carers as a peer mentor to foster interpersonal relationships.

Equal relationships between mentors and mentees are indicated to be important, with Schwartz and Levin (2022) suggesting the role of modelling to enable positive outcomes in peer‐delivered interventions. Our results imply that where this modelling was possible, such as mentors prioritising and recognising the importance of self‐care and thus encouraging this to mentees, mentors perceived positive outcomes facilitated by the mentoring relationship, such as increased self‐confidence. Several mentors highlighted this recognition and modelling of prioritising self‐care and the knock‐on effects as one of the most important elements of their mentoring role. This was an unintended and unexpected outcome for peer mentors, and it would be interesting to further consider whether these unexpected outcomes are commonplace in future studies that involve peer mentoring.

Previous literature has highlighted that emotional difficulties can arise within a peer support role (Shilling et al. 2013) alongside difficulties in maintaining personal well‐being (Ahmed et al. 2015). These negative findings were noticeably absent within our data and, whilst there are limitations due to the sample size, it is worth considering whether the reported positive impacts of concordantly undertaking the online mindfulness intervention expressed by our participants may have enabled mentors to better have the tools to mitigate these potential negative impacts. Our study did not have the data to interrogate this possibility as this question was not directly asked. However, in future research where peer mentors are undertaking a well‐being intervention before supporting others, data could be collected to examine this.

It is worth reflecting on parent peer mentor expressions that whilst many had not been in contracted employment, they had been working within the home, needing to sacrifice their paid roles due to the extent of demands within their caring role. Gender roles may be more pronounced in caring for people with intellectual and developmental disabilities, and this lack of remuneration and recognition for the magnitude of caring labour may subsequently disproportionately impact mothers, potentially evident within our parental sample.

### 4.1 Strengths and Limitations

A strength of this study was that we interviewed parents and adult siblings who had been employed as peer mentors. We aimed to recruit a representative mentoring group, but notably absent were male family carers, with no father peer mentors being employed on the project and only one brother peer mentor being interviewed. This highlights the need to explore further the barriers to representing males within this type of research. Additionally, we did not gather wider demographic data about peer mentors, including ethnicity, age, employment status, and so we cannot consider how any of these characteristics may impact on their experience of being a peer mentor. This is an area that future researchers should consider.

Although all peer mentors invited to interview took part, five peer mentors over the course of the project had circumstances which prevented them from continuing in the role. Sampling bias is, therefore, a limitation of the study as, whilst the early withdrawal of mentors who did not undertake the role would not have been able to offer insight into the experiences of being a peer mentor as they did not undertake the role, we did not speak with anybody who disengaged with the project and thus have limited understanding of the barriers and reasons for disengagement. We may not have captured the full range of experiences of all mentors by including those unable to continue in their role; this may also reflect the predominantly positive findings reported by mentors.

### 4.2 Implications for Practice

Mentors have many demands in their own lives, and the mentoring role was intended to provide an opportunity for family carers to be paid to utilise their own experiences in a manner that would allow them to work flexibly and practically around these demands. The peer mentor role aimed to enhance equity for those facing barriers to employment. However, providing flexible roles also meant providing remote and potentially isolating roles for peer mentors. Whilst no demographic data were collected on the ethnicity of mentors, during interviews, three mothers highlighted that their intersectionality of also belonging to ethnic minority communities increased their sense of isolation and difficulty in locating other parents in the same position. It is worth considering that aspects of the process that could lead to peer mentors not feeling as integrated within their cohort, such as missed training, may have been felt more pertinently by those already feeling isolated; one peer mentor shared that they felt less confident assimilating into informal group conversations such as those through WhatsApp due to this.

A key learning point was the emphasis on the importance of scaffolding the process. Future projects could consider organised and structured contact points for mentors to meet with one another during the process to allow for those who may have needed flexibility during the training to develop confidence with their peers and enable them to participate more widely within the group. This contact would allow greater opportunity for connection, for the group to increase mentor cohesiveness, and to reduce the risk of isolation and potential negative consequences of remote roles.

It is important to acknowledge the importance of clearly defining the peer support role and its parameters from the offset, possibly pre‐empting some of the difficulties throughout the training process. A lack of complete understanding of the duality of simultaneously being a peer and peer mentor could impact on the realisation of the full potential of the role and undermine the motivations of the mentors. A potential way to mitigate this would be to rename the role to ‘coach’ or a similar term to delineate the role as that of facilitating goals rather than forming in‐depth relationships.

## Ethics Statement

Ethical approval was granted by the University of Warwick's Humanities and Social Sciences Research Ethics Committee (reference 101/21‐22).

## Conflicts of Interest

D.M. recruited and employed the peer mentors through their role at the Foundation for People with Learning Disabilities. We have no other conflicts of interest to disclose.

## Data Availability

Data supporting this study cannot be made available due to ethical restrictions.

## References

[jar70102-bib-0001] Adams, W. E. 2020. “Unintended Consequences of Institutionalizing Peer Support Work in Mental Healthcare.” Social Science & Medicine 262: 113249. 10.1016/j.socscimed.2020.113249. 32768773

[jar70102-bib-0002] Ahmed, A. O. , K. M. Hunter , A. P. Mabe , S. J. Tucker , and P. F. Buckley . 2015. “The Professional Experiences of Peer Specialists in the Georgia Mental Health Consumer Network.” Community Mental Health Journal 51, no. 4: 424–436. 10.1007/s10597-015-9854-8. 25724917

[jar70102-bib-0003] Arnold, C. K. , T. Heller , and J. Kramer . 2012. “Support Needs of Siblings of People With Developmental Disabilities.” Intellectual and Developmental Disabilities 50, no. 5: 373–382. 10.1352/1934-9556-50.5.373. 23025639

[jar70102-bib-0004] Bjornstad, G. , B. Cuffe‐Fuller , O. C. Ukoumunne , et al. 2021. “Healthy Parent Carers: Feasibility Randomised Controlled Trial of a Peer‐Led Group‐Based Health Promotion Intervention for Parent Carers of Disabled Children.” Pilot and Feasibility Studies 7: 144. 10.1186/s40814-021-00881-5. 34301334 PMC8298691

[jar70102-bib-0005] Bourke‐Taylor, H. M. , D. A. Lee , L. Tirlea , K. Joyce , P. Morgan , and T. P. Haines . 2021. “Interventions to Improve the Mental Health of Mothers of Children With a Disability: Systematic Review, Meta‐Analysis and Description of Interventions.” Journal of Autism and Developmental Disorders 51, no. 10: 3690–3706. 10.1007/s10803-020-04826-4. 33389452

[jar70102-bib-0031] Braun, V. , and V. Clarke . 2013. Successful Qualitative Research: A Practical Guide for Beginners. Sage.

[jar70102-bib-0006] Brennan, D. , M. D'Eath , P. McCallion , and M. McCarron . 2023. “Health and Well‐Being of Sibling Carers of Adults With an Intellectual Disability in Ireland: Four Waves of Data.” British Journal of Learning Disabilities 51: 534–543. 10.1111/bld.12532.

[jar70102-bib-0007] Case, S. 2000. “Refocusing on the Parent: What Are the Social Issues of Concern for Parents of Disabled Children?” Disability & Society 15, no. 2: 271–292. 10.1080/09687590025676.

[jar70102-bib-0010] Coulman, E. , N. Gore , G. Moody , et al. 2021. “Early Positive Approaches to Support (E‐PAtS) for Families of Young Children With Intellectual Disability: A Feasibility Randomised Controlled Trial.” Frontiers in Psychiatry 12: 1–17. 10.3389/fpsyt.2021.729129. PMC8725992 34992552

[jar70102-bib-0009] Coulman, E. , N. Gore , G. Moody , et al. 2022. “Early Positive Approaches to Support for Families of Young Children With Intellectual Disability: The E‐PAtS Feasibility RCT.” Public Health Research 10, no. 2: 1–144. 10.3310/HEYY3556. 35129939

[jar70102-bib-0012] Flynn, S. , R. P. Hastings , C. Burke , et al. 2020. “Online Mindfulness Stress Intervention for Family Carers of Children and Adults With Intellectual Disabilities: Feasibility Randomized Controlled Trial.” Mindfulness 11: 2161–2175. 10.1007/s12671-020-01436-0.

[jar70102-bib-0013] Fortuna, K. L. , A. L. Myers , J. Ferron , et al. 2022. “Assessing a Digital Peer Support Self‐Management Intervention for Adults With Serious Mental Illness: Feasibility, Acceptability, and Preliminary Effectiveness.” Journal of Mental Health 31, no. 6: 833–841. 10.1080/09638237. 35088619 PMC9329481

[jar70102-bib-0014] Fortuna, K. L. , J. A. Naslund , J. M. LaCroix , et al. 2020. “Digital Peer Support Mental Health Interventions for People With a Lived Experience of a Serious Mental Illness: Systematic Review.” JMIR Mental Health 7, no. 4: e16460. 10.2196/16460. 32243256 PMC7165313

[jar70102-bib-0015] Hammarberg, K. , G. Sartore , W. Cann , and J. R. W. Fisher . 2014. “Barriers and Promoters of Participation in Facilitated Peer Support Groups for Carers of Children With Special Needs.” Scandinavian Journal of Caring Sciences 28, no. 4: 775–783. 10.1111/scs.12110. 24405486

[jar70102-bib-0016] Haslam, C. , T. Cruwys , S. A. Haslam , G. Dingle , and M. X. Chang . 2016. “Groups 4 Health: Evidence That a Social‐Identity Intervention That Builds and Strengthens Social Group Membership Improves Mental Health.” Journal of Affective Disorders 194: 188–195. 10.1016/j.jad.2016.01.010. 26828756

[jar70102-bib-0017] Hennink, M. , and B. N. Kaiser . 2021. “Sample Sizes for Saturation in Qualitative Research: A Systematic Review of Empirical Tests.” Social Science & Medicine 292: 114523. 10.1016/j.socscimed.2021.114523. 34785096

[jar70102-bib-0019] Ritchie, J. , and L. Spencer . 1994. “Qualitative Data Analysis for Applied Policy Research.” In Analyzing qualitative data, edited by A. Bryman and R. Burgess , 305–329. Routledge. 10.4324/9780203413081_chapter_9.

[jar70102-bib-0020] Schwartz, A. E. , and M. Levin . 2022. “Feasibility of a Peer Mentoring Programme for Young Adults With Intellectual and Developmental Disabilities and Co‐Occurring Mental Health Conditions.” British Journal of Learning Disabilities 50, no. 3: 433–445. 10.1111/bld.12396.

[jar70102-bib-0021] Shilling, V. , S. Bailey , S. Logan , and C. Morris . 2015a. “Peer Support for Parents of Disabled Children Part 1: Perceived Outcomes of a One‐To‐One Service, a Qualitative Study.” Child: Care, Health and Development 41, no. 4: 524–536. 10.1111/cch.12223. 25521697

[jar70102-bib-0022] Shilling, V. , S. Bailey , S. Logan , and C. Morris . 2015b. “Peer Support for Parents of Disabled Children Part 2: How Organizational and Process Factors Influenced Shared Experience in a One‐To‐One Service, a Qualitative Study.” Child: Care, Health and Development 41, no. 4: 537–546. 10.1111/cch.12222. 25556621

[jar70102-bib-0024] Shilling, V. , C. Morris , J. Thompson‐Coon , O. Ukoumunne , M. Rogers , and S. Logan . 2013. “Peer Support for Parents of Children With Chronic Disabling Conditions: A Systematic Review of Quantitative and Qualitative Studies.” Developmental Medicine and Child Neurology 55, no. 7: 602–609. 10.1111/dmcn.12091. 23421818

[jar70102-bib-0025] Simpson, A. , C. Oster , and E. Muir‐Cochrane . 2018. “Liminality in the Occupational Identity of Mental Health Peer Support Workers: A Qualitative Study.” International Journal of Mental Health Nursing 27, no. 2: 662–671. 10.1111/inm.12351. 28548455 PMC5900877

[jar70102-bib-0026] Suitor, J. J. , K. Pillemer , and S. Keeton . 1995. “When Experience Counts: The Effects of Experiential and Structural Similarity on Patterns of Support and Interpersonal Stress.” Social Forces 73, no. 4: 1573–1588. 10.2307/2580459.

[jar70102-bib-0027] Theurer, K. A. , R. I. Stone , M. J. Suto , V. Timonen , S. G. Brown , and W. B. Mortenson . 2022. “‘It Makes You Feel Good to Help!’: An Exploratory Study of the Experience of Peer Mentoring in Long‐Term Care.” Canadian Journal on Aging/La Revue Canadienne du Vieillissement 41, no. 3: 451–459. 10.1017/S0714980821000611. 35538870

[jar70102-bib-0028] Veith, E. M. , J. E. Sherman , T. A. Pellino , and N. Y. Yasui . 2006. “Qualitative Analysis of the Peer‐Mentoring Relationship Among Individuals With Spinal Cord Injury.” Rehabilitation Psychology 51, no. 4: 289–298. 10.1037/0090-5550.51.4.289.

[jar70102-bib-0029] Whiteman, K. L. , J. A. Naslund , E. A. DiNapoli , M. L. Bruce , and S. J. Bartels . 2016. “Systematic Review of Integrated General Medical and Psychiatric Self‐Management Interventions for Adults With Serious Mental Illness.” Psychiatric Services 67, no. 11: 1213–1225. 10.1176/appi.ps.201500521. 27301767 PMC5089924

[jar70102-bib-0030] Whitmore, J. 1996. Coaching for Performance. Nicholas Brealey.

